# Ecological effects on female bill colour explain plastic sexual dichromatism in a mutually-ornamented bird

**DOI:** 10.1038/s41598-021-93897-z

**Published:** 2021-07-22

**Authors:** Rita Freitas, Cristiana Marques, Gonçalo C. Cardoso, Sandra Trigo

**Affiliations:** CIBIO/InBIO, Centro de Investigação em Biodiversidade e Recursos Genéticos da Universidade do Porto, 4485-661 Vairão, Portugal

**Keywords:** Behavioural ecology, Social evolution

## Abstract

Sex differences in ornamentation are common and, in species with conventional sex roles, are generally thought of as stable, due to stronger sexual selection on males. Yet, especially in gregarious species, ornaments can also have non-sexual social functions, raising the possibility that observed sex differences in ornamentation are plastic. For example, females may invest in costly ornamentation more plastically, to protect body and reproductive ability in more adverse ecological conditions. We tested this hypothesis with experimental work on the mutually-ornamented common waxbill (*Estrilda astrild*), supplementing their diets either with pigmentary (lutein, a carotenoid) or non-pigmentary (vitamin E) antioxidants, or alleviating winter cold temperature. We found that both lutein and vitamin E supplementation increased red bill colour saturation in females, reaching the same mean saturation as males, which supports the hypothesis that female bill colour is more sensitive to environmental or physiological conditions. The effect of vitamin E, a non-pigment antioxidant, suggests that carotenoids were released from their antioxidant functions. Alleviating winter cold did not increase bill colour saturation in either sex, but increased the stability of female bill colour over time, suggesting that female investment in bill colour is sensitive to cold-mediated stress. Together, results show that waxbill bill sexual dichromatism is not stable. Instead, sexual dichromatism can be modulated, and even disappear completely, due to ecology-mediated plastic adjustments in female bill colour.

## Introduction

In animals with conventional sex roles, reproduction in females is limited by their physiology, while males can differ greatly in reproductive success depending on the number of mates and fertilizations and, thus, experience strong sexual selection^1,2^. This explains why it is common that sexual ornamentation is present only in males or, in mutually-ornamented species, that males are the more ornamented sex^1,3–5^. Sex differences in ornamentation are thought to often have a strong genetic basis^6–8^, anchored for example in hormonal differences between males and females^9^. This view does not imply that male and female ornaments do not change, but only that on average the sex difference remains present in populations even if there is plasticity in the expression of male and/or female ornamentation.

Especially in gregarious animals, ornamentation can be involved in several aspects of non-sexual social selection, such as competition among group members for resources or social hierarchy^10,11^. Unlike sexual selection, social selection in species with conventional sex roles need not to be stronger for males than females. Therefore, some sex differences in ornamentation may not be stable. The phenomenon of plastic sex differences in ornamentation, i.e., those differences that result from sex differences in phenotypic plasticity, is poorly documented or investigated in species with conventional sex roles. Some examples were nonetheless reported, such as the ornamental bill colour of some birds, which can change plastically^12–15^ and where changes in colour appear affected by ecology or physiology differently in females and in males^16–18^.

Plastic sex differences in ornamentation are predicted to evolve when ornamentation benefits both sexes, but females and males differ in whether or how they benefit from adjusting ornamentation in response to external or physiological conditions^19,20^. Ornamentation is often costly, and investing in ornaments may imply trade-offs with investing in physiological functions^21^. For example, investing in carotenoid-based colour ornaments may compromise allocating carotenoids for antioxidant functions^22,23^, and may be limited by individual differences in coping with oxidative stress^24^, immunocompetence^25^ or parasites^26^. Since investment in ornamentation can imply trade-offs with other functions, sex differences in life-history could cause females and males to adjust investment in ornaments differently, giving rise to sex differences in the phenotypic plasticity of ornamentation.

**Figure 1:**
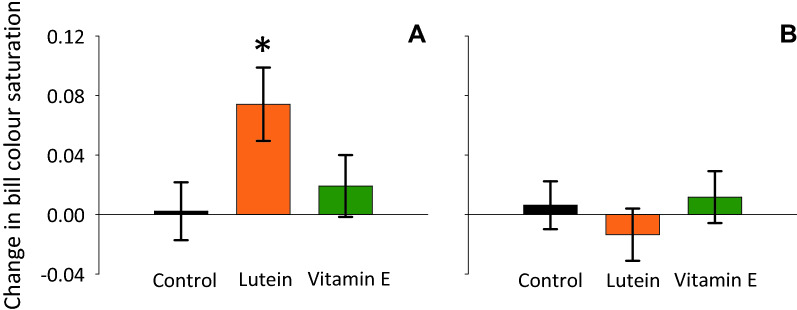
Changes in (**A**) female and (**B**) male bill colour saturation (mean ± SE) from before to after each diet manipulation (control in black, lutein-supplemented in orange, and vitamin E-supplemented in green). Asterisks mark statistically significant differences in post-hoc tests against the control treatment.

The common waxbill (*Estrilda astrild*) is a gregarious and mutually-ornamented bird with conventional sex roles^27^, where both sexes have red plumage in the breast and face mask, and a vividly red bill that coins the species common name (waxbill, in reference to red sealing wax). Waxbill plumage and bill colour ornamentation influence social preferences in laboratory tests, similarly in males and females^29^, and in the wild males in better body condition on average have more saturated red bill colour^30^. Studying bill colour in common waxbills, Funghi et al.^18^ suggested that female ornamentation may be more sensitive to the quality of environmental conditions, perhaps because females need to balance investment in ornamentation with maintaining physiological condition for reproduction, and also suggested that female adjustments investing in ornaments may explain plastic sex differences in ornamentation. Males, on the contrary, perhaps because of not bearing a large physiological cost of reproduction, would invest in ornamentation across a wider range of ecological conditions. This hypothesis is partly supported by three observations on common waxbill ornamentation:

1. Although, on average, males are more ornamented than females, with more saturated red colour in the bill and plumage^28^, the range of among-individual variation in ornamentation and in bill colour saturation is extensive and overlaps between males and females (see Figure 1F in^29^), and the sex difference in bill colour can disappear in particularly favourable ecological conditions (ad libitum food and mild temperatures^18,29^).
2. Bill colour saturation is not a badge of status signalling social dominance, since neither bill colour nor changes in colour reflect aggressiveness or, as a consequence, dominance^18^ (see also^31^).
3. Instead, a correlational test, using natural temperature fluctuations during 7 months comprising spring and summer, found that changes in the bill colour saturation of females, but not males, were positively related to temperature, especially night temperatures that can pose the greatest energetic stress to small birds^18^.

These observations suggest that female and male waxbills do not differ in the maximum bill colour saturation that they express, but that females plastically adjust investment in bill colour depending on the quality of environmental conditions, and perhaps this is what causes sexual dichromatism. However, the critical observation that female bill colour saturation responds to environmental quality (observation 3, above) is only correlational, and needs experimental confirmation.

Here, we use female and male common waxbills in experiments that manipulate either diet or temperature, to test how these environmental conditions affect investment in bill colour saturation. We study bill colour saturation because it can reflect carotenoid availability^32^, because saturation is a colour parameter showing sexual dichromatism in waxbill bills^28^, and because previous observations suggest that sex differences in colour saturation change depending on the environment^18,29^.

First, we manipulated dietary availability of a carotenoid and a non-carotenoid antioxidant: lutein and vitamin E, respectively. Lutein is the main dietary carotenoid found in waxbills blood^33^, and we hypothesize that increasing its availability allows more investment in bill colour saturation. Vitamin E is a powerful non-colorant antioxidant^34^, and increasing its availability can be hypothesized to have positive effects on coloration (e.g., *Larus michahellis*^35^) by alleviating the use of carotenoid pigments for antioxidant functions^36^. We predict that these effects should be more pronounced in females, if females are the sex whose investment in ornamentation is most affected by environmental conditions and availability of resources. Second, we manipulated temperature during winter, to test if alleviating the energetic stress of winter cold, especially cold nights, facilitates investment in bill colour. Again, we predict that effects of protecting from cold should be more pronounced in females, if female investment in bill colour is most affected by environment-mediated energetic constrains.

## Results

### Diet experiment

For female bill colour saturation, we found a significant interaction between diet treatment (control, lutein and vitamin E) and time (i.e., at the onset vs. during each diet manipulation; F_2,66_ = 3.37, P = 0.04, Fig. 1A; effects of treatment and time: F_2,66_ = 1.9, P = 0.15, and F_1,66_ = 2.91, P = 0.09, respectively), indicating that changes in bill colour saturation differed between treatments. Post hoc tests showed that changes in female bill colour saturation differed between the control treatment and lutein supplementation (interaction effect: F_1,44_ = 4.71, P = 0.035; Table S1) and not vitamin E supplementation (interaction effect F_1,44_ = 0.77, P = 0.77; Table S1). We found no effects of the diet manipulation in male bill colour (interaction between treatment and time: F_2,60_ = 0.05, P = 0.86, Fig. 1B; effects of treatment and time: F_2,60_ = 1.88, P = 0.16, and F_1,60_ = 0.03, P = 0.86).

Changes in body weight did not differ significantly between diet treatments in females (interaction between treatment and time: F_2,65_ = 0.22, P = 0.80; effects of treatment and time: F_2,65_ = 0.16, P = 0.85 and F_1,65_ = 2.82, P = 0.10) nor in males (interaction: F_2,60_ = 0.10, P = 0.91; effects of treatment and time: F_2,60_ = 0.02, P = 0.98, and F_1,60_ = 0.70, P = 0.41).

**Figure 2:**
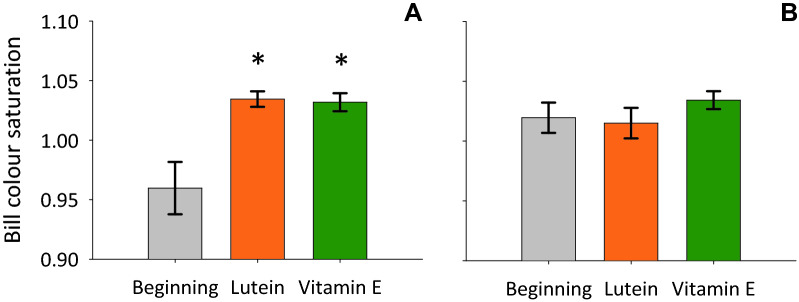
(**A**) Female and (**B**) male bill colour saturation (mean ± SE) before any diet manipulation (grey), and after each 4-week diet supplementation treatment (colours as in Fig. 1). Asterisks mark statistically significant differences between measurements after diet supplementation and measurements before any diet manipulation.

It is possible that finding an effect of lutein, but not of vitamin E, on changes in female bill colour (Fig. 1A) could be due to carry-over effects because, while the lutein treatment followed the control diet, the vitamin E treatment often followed the lutein treatment (and therefore its initial bill colour measurement could already be elevated). To test effects of the diet treatment in a manner that is more robust to carry-over effects, we compared initial measurements of bill colour saturation, before any treatment (grey bars in Fig. 2), with measurements after each diet-supplementation treatments (orange and green bars in Fig. 2). We found that bill colour saturation differed significantly in females (F_2,33_ = 9.25, P = 0.001; Table S2). Pairwise post-hoc comparisons with the measurements before any treatment showed that bill colour saturation was higher in the lutein (F_1,22_ = 10.59, P = 0.004) and the vitamin E treatments (F_1,22_ = 9.62, P = 0.005; Fig. 2A). Again we found no effects of the diet treatments on male bill colour saturation (F_2,30_ = 0.80, P = 0.46; Fig. 2B).

### Temperature manipulation

**Figure 3:**
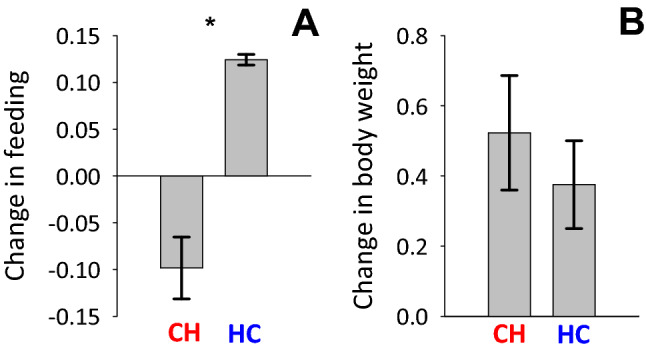
Changes (mean ± SE) in (**A**) feeding and (**B**) body weight (g) during the two temperature treatments. *CH* cold–hot treatment, *HC* hot–cold treatment. Asterisks mark statistically significant differences.

Changes in feeding differed between the two temperature treatments (t = − 6.64; N = 6 cages; P = 0.003), with birds in the cold–hot treatment (i.e., 4 weeks in ambient temperature, and then 4 weeks 6ºC above ambient temperature) decreasing feeding and birds in the hot–cold treatment (i.e., reverse order of temperature manipulation) increasing feeding (Fig. 3A). Changes in body weight did not differ between the cold–hot and hot–cold treatments (t = 0.73; N = 23 birds; P = 0.48; Fig. 3B).

**Figure 4:**
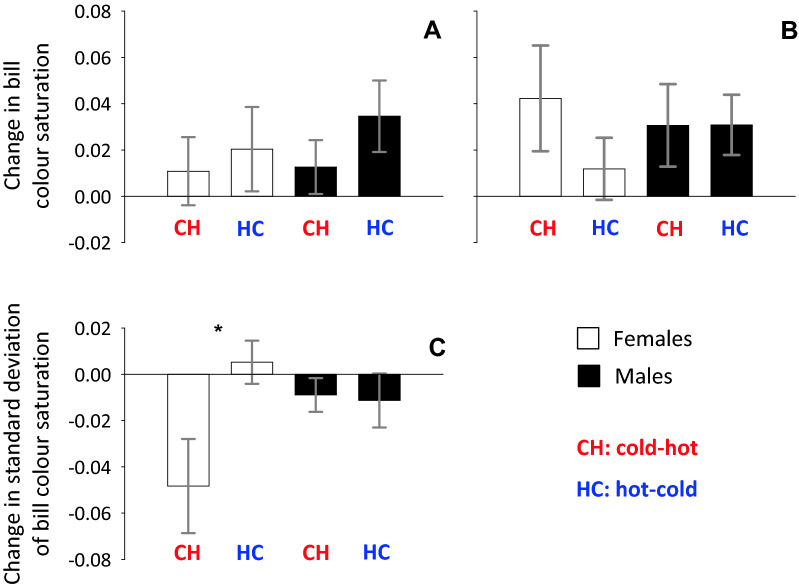
Changes in bill colour saturation (means ± SE) using data from (**A**) the entire 4-week periods of temperature manipulations, or (**B**) the last 2 weeks of each 4-week period temperature manipulations. (**C**) Changes in the standard deviation of bill colour saturation. *CH* cold–hot treatment, *HC* hot–cold treatment. Asterisks mark statistically significant differences between treatments.

Changes in bill colour saturation from the first 4 weeks to the last 4 weeks of the temperature manipulation experiment, using data from the entire 4-week periods, did not differ between the cold–hot and the hot–cold treatments in females (t = − 0.40; N = 11 birds; p = 0.70; white bars in Fig. 4A) nor in males (t = − 1.14; N = 12 birds; p = 0.28; black bars in Fig. 4A). In case bill colour takes a long time adjusting to temperature, we repeated this comparison using only data from the last 2 weeks of each 4-week period of temperature manipulation. Changes in female bill colour saturation in the cold–hot treatment were now ca. 4 times higher than in the hot–cold treatment, but among-individual variation in these colour changes was large and the difference between treatments was non-significant (t = 1.20; N = 11; P = 0.26; white bars in Fig. 4B). In males, the extent of bill colour changes did not differ in the cold–hot and the hot–cold treatments (t = 0.01; N = 12; P = 0.99; black bars in Fig. 4B).

Within-individual fluctuation in female bill colour, computed as the standard deviation of colour saturation in each 4-week period, tended to decrease in the cold–hot treatment and to slightly increase in the hot–cold treatment, and the difference between treatments was significant (t = − 2.54; N = 11 birds; P = 0.03, white bars in Fig. 4C). In males, changes in the extent of fluctuations in bill colour did not differ between treatments (t = 0.17; N = 12 birds; P = 0.87; black bars in Fig. 4C).

## Discussion

Results from both our experiments support the hypothesis that female bill colour is more sensitive to environmental conditions than male bill colour. While we found no effects of lutein- or vitamin E supplementation on male bill colour saturation, female bill colour increased with lutein supplementation and, compared to colour saturation prior to any diet manipulation, also with vitamin E supplementation. In the temperature experiment, we again found no effects on male bill colour saturation or on its stability over time. In females, while we also found no differences in mean bill colour, bill colour saturation became more stable when increasing winter temperature compared to when decreasing temperature, suggesting that female bill colour is sensitive to temperature-mediated energetic stress.

Previous experiments with different species have shown that dietary carotenoid supplementation can increase the expression of carotenoid-based colour ornamentation in bills (e.g.^3,16,37,38^) and other bare parts (e.g.^39–41^) as well as in plumage (e.g.^14,32,42–44^). This has been interpreted as due to carotenoids being obtained exclusively from the diet (and not synthesized by animals^45^), which could limit carotenoid availability for coloration^46,47^ (but see^48^), especially because carotenoids may be required for different functions, including pigmentation and antioxidant functions^23,49,50^ (but see^51^). Similarly to work on other species, we found that carotenoid (lutein) supplementation in the diet can enhance the expression of carotenoid-based ornamentation in waxbills. Interestingly, we found this effect of carotenoid supplementation on bill colour only in females, and not in males, providing experimental evidence for the suggestion that, in the common waxbill, female investment in ornamentation is more sensitive to environmental conditions than male investment in ornamentation^18^. As a consequence of this effect on females, the bill sexual dichromatism normally observed in waxbills^28,29^ and observed here at the onset of the experiment (grey bars in Fig. 2), disappeared after dietary carotenoid supplementation (orange bars in Fig. 2). This shows that bill sexual dichromatism in the common waxbill is plastic, and its plasticity can be mediated by ecological effects on female bill colour. To our knowledge, only one other study on a mutually-ornamented species, the black-legged kittiwake (*Rissa tridactyla*), reported such an effect of carotenoid supplementation on sexual ornamentation: carotenoid supplementation was associated with redder carotenoid-based colour in the gape and tongue of females, but not males^52^ (see also^53^ for a study in chicks of the common tern, *Sterna hirundo*).

We also found evidence that dietary supplementation of vitamin E, a non-pigmentary antioxidant, enhanced the expression of bill colour saturation, again in females and not males. Vitamin E is the main lipophilic antioxidant in animals, like carotenoids it is only acquired from the diet, and its abundance can significantly affect the antioxidant capacity of individuals and affect immune protection^34,54^. Finding that vitamin E supplementation can increase carotenoid-based colour ornamentation, which has been reported in other species as well (birds^35,55,56^; reptiles^57^; fish^58^), supports the hypothesis that abundant non-pigment antioxidant molecules free carotenoids from antioxidant functions and make them available for coloration, such that carotenoid-based ornamentation could signal antioxidant capacity^22,23,36^. Again, finding an effect of vitamin E supplementation in female bill colour, but not male, supports the hypothesis that female waxbills require better physiological conditions to invest in costly ornamentation, while males prioritize investment in ornamentation in worse conditions.

Our temperature manipulations, exposing waxbills to winter temperatures and then increasing temperature by ca. 6 °C, or vice-versa, were physiologically meaningful because alleviating winter cold was associated with less feeding early in the morning. These birds were exposed to natural winter temperatures, including cold nights, near the less equatorial limit of the waxbill geographic distribution^59^ and therefore they experienced very cold conditions within the natural range of temperatures for the species. Winter cold, especially during nights, is an important energetic stressor for small birds, but our waxbills had ad libitum food and there were no changes in body mass associated with the temperature treatments (nor with the previous diet treatments), indicating that experimental treatments were not damaging to their body condition. Based on results from a previous correlational study with waxbills^18^, we expected that higher temperatures increased investment in bill colour saturation, at least in females. Earlier work with male zebra finches (*Taeniopygia guttata*) had also reported that exposure to cold diminish bill colour saturation^16^.

Contrary to expectations, we found no significant effects of temperature manipulations on the mean bill colour saturation of either female or male waxbills. When comparing bill colour during the last 2 weeks of temperature manipulations, to allow more time for bill colour to adjust to temperature, we did find that mean changes in female bill colour saturation were much higher (ca. four-fold higher) when passing from ambient to warmer temperatures than in the reverse treatment, but among-individual variation in the extent of these changes was large and the difference was non-significant. The explanation for the different results here and in Funghi et al.^18^ or Eraud et al.^16^ is not clear. It may be related with the time of the year in which the different studies were conducted, since our experiments were made in winter and the previous correlational study with waxbills was conducted across spring and summer^18^. Spring and summer correspond to the breeding season of waxbills in this region^60^, which may alter various aspects of their biology. Or the different results may be due to the extent of temperature differences used, since we alleviated winter cold with a 6 °C warming, while the range of natural temperature differences the previous correlational study was higher, and Eraud et al.^16^ also subjected experimental birds to a much higher temperature difference of 20 °C.

We did find, however, a positive effect of alleviating winter cold on female bill colour, in the form of more stable bill colour saturation. Repeated measurements of waxbill bill colour show considerable fluctuation through time^18^, perhaps reflecting changing investment in colour ornamentation. Since the avian bill is a living tissue and its colour may be condition-dependent (e.g.^12,15,16^) fluctuations in colour are expected to occur, for example, if challenges to individual conditions render them less able to sustain a stable investment in bill colour. For example, energetic stress caused by cold temperatures can cause oxidative stress^61–64^ (but see^65^) which might require the mobilization of carotenoids for antioxidant^22,23,49^ or other physiological functions^25,26^. Therefore, bill colour fluctuations may be symptomatic of unstable individual condition. We found that females passing from ambient to warmer temperatures reduced the degree of fluctuation in bill colour saturation, as compared with the reverse treatment, but in males there were no effects of the temperature manipulation on colour stability. This effect of warmer temperatures on the stability of female bill colour saturation supports the hypothesis that female bill colour is more sensitive to environmental conditions, albeit in a different manner than suggested by previous correlational work^18^.

Taken together, our results provide experimental evidence for various ecological effects on the bill colour of female common waxbills. Dietary enrichment with either a carotenoid or a non-carotenoid antioxidant increased female but not male bill colour saturation, and protection from winter cold increased the stability of female but not male bill colour saturation. It was previously suggested that female ornamentation may be sensitive to environmental conditions because of female life-history and reproductive physiology^18^. According to this view, bill colour in American goldfinches (*Spinus tristis*) was found to reflect individual differences in female immune function but not in males, who consistently invest in bill coloration irrespective of immune function^17^, and a recent field study with pied flycatchers (*Ficedula hypoleuca*) found that changes in female but not male plumage ornaments were affected by climate during breeding, such that cold and rainy breeding seasons decreased female ornamentation, likely reflecting a greater cost of breeding in those years^66^.

The ecology-mediated changes in the bill colour of female waxbills that we showed here strongly affect the extent of sexual dichromatism, and can go as far as eliminating the sexual dichromatism that is usually observed in this species^28,29^. This finding departs from the usual view that, in species with conventional sex roles, sex differences in ornamentation have a strong component of genetic determination, due to stronger sexual selection on males, and therefore sexual dichromatism should remain present despite a certain degree of plasticity in ornamental colours. Instead, our results suggest that sex differences in life-history and/or the strength of sexual selection may cause sex differences, not in the degree of ornamentation, but on the extent that investment in ornamentation is plastic and dependent on ecological conditions.

## Methods

### Diet experiment

#### Housing and diet manipulations

This experiment took place in a research aviary at the Research Centre in Biodiversity and Genetic Resources (CIBIO-InBio), University of Porto, Portugal, and lasted 14 weeks, from the 23rd July until the 29th October 2018, which is still part of the waxbill breeding season in this region^60^. Common waxbills (*Estrilda astrild*) were acquired from certified breeders in March 2018, approximately 4 months before the experiment. We used 23 birds, 11 males and 12 females, all ringed with individually numbered metal rings and housed in mixed-sex groups in six metal cages (88.5 × 30 × 40 cm). Each cage housed two males and two females (except one cage with one male and two females). The cages were in an outdoor aviary, sheltered from wind and direct sunlight, and with natural illumination supplemented with artificial lights in the same dial cycle as the natural light. One of the aviary walls was made of metal grid so that the temperature and humidity inside the aviary were identical to outside conditions. Each cage had four long perches, sand in the floor, ad libitum water in two dispensers (changed every other day) and food (Tropical Finches Prestige, Versele-Laga) in two long feeders that allowed all birds to feed simultaneously. A bird bath filled with water was placed inside the cages for birds to bath twice a week. All experimental protocols were carried out in accordance with ARRIVE guidelines and regulations, Helsinki guidelines and were approved by the ORBEA (Organism for Animal Welfare) of CIBIO-InBIO (ethics assessment # ORBEA_2019_Estrilda).

During the 14 weeks of the experiment, we performed diet manipulations in a balanced manner across cages. Each of three different treatments—control, lutein-supplemented and vitamin E-supplemented—was administered to two bird cages for 4 weeks, then there was an interval of 1 week with standard control diet, for the following 4-weeks period the treatments were rotated (cages that had had control diets now were lutein-supplemented, those that had had lutein now were vitamin E-supplemented, and those that had had vitamin E now had control diets) and, after another 1-week interval, for the last 4-weeks period the treatments rotated again, so that all cages experienced the three diet treatments in balanced dates.

We manipulated carotenoid and the vitamin E content by adding either lutein or vitamin E to the drinking water, while in control diets nothing was added to the water. Concentrations were 0.4 g/l of lutein (FloraGlo), and 1.8 g/l of vitamin E (A.C. Grace, Unique E Natural Vitamin E Oil). Since common waxbills do not drink much water^67^ (we estimate drinking on the order of magnitude of 0.5 ml per day), and following experimental methods for other similar bird species, we chose the above dosages so as to administer ca. 0.2 mg lutein per day per bird^43,68^ and ca. 0.9 mg vitamin E per day per bird^35,43^. By comparison, the amount of carotenoid and vitamin E obtained from the commercial mixture of seeds should be very small. Birds had ad libitum access to a commercial mixture of seed with the following composition: canary seed 40%, panicum yellow 28%, yellow millet 15%, Japanese millet 10%, panicum red 6% and perilla seed 1% (Tropical Finches, Australian Finches, Versele-Laga). The most abundant seed in this mixture, canary seed, has a low carotenoid concentration of 2.8 µg/g^69^ and a low antioxidant capacity^70^. Throughout the 4-week periods of diet manipulations, food and water were replenished every other day to ensure fresh supplements.

#### Bill colour measurements and body weight

We measured bill colour saturation a total of fifteen times during the experiment, starting in the first day of experiments, on the 23rd July, and then weekly until the end of experiments, on the 29th October 2018. We used reflectance spectrophotometry, with an Ocean Optics usb4000 spectrophotometer coupled to a PX-2 xenon light source, and calibrated 0% and 100% reflectance of measurements with an opaque black velvet and an Ocean Optics WS-1-SL white standard, respectively. Measurements were made with the reflectance probe gently touching perpendicularly the bill surface, and then enveloping the area with black velvet to prevent light contamination from outside the probe. Each time we measured four spectra from the bill, two on each side of the upper mandible, because the upper mandible is the most visible part of the bill. We weighed birds with a spring balance to the nearest 0.05 g at the beginning and the end of each 4-week period of the experiment, at the same time of the colour measurements, in the mornings.

We changed reflectance below 1% to 1%, because small measurement inaccuracies near 0% reflectance are large in relative terms, and then log_10_-transformed reflectance spectra to quantify reflectance in a ratio scale, which conforms to mechanisms of colour production and perception^71^. As in Funghi et al.^18^, we computed bill colour saturation by averaging log_10_-reflectance across the wavelengths at which the reflectance of red bills plateaus and is highest (600 to 700 nm), and then subtracting the average log_10_-reflectance across the entire bird-visible range of wavelengths (320–700 nm^72^; note that differences of logarithms quantify ratios of raw measurements). We averaged colour saturation for the four bill spectra of each bird, in each day.

#### Statistical analyses

During the 4-week periods of diet supplementations, we observed that bill colour saturation on average showed a plateau after week 2 (Figure S1). Therefore, in statistical models we used as dependent variable the mean colour saturation in weeks 2–4 from each 4-week period, as the measure of colour during each diet manipulation. For each sex, we ran General Mixed Models (GLMM) with bill colour saturation as dependent variable, individual identity as random factor, diet treatment as a 3-level factor (control, lutein, vitamin E), time as a 2-level factor (at the onset vs. during each diet manipulation), and the interaction between diet treatment and the two-level time factor. The result of interest is the interaction effect, which tests if changes of bill colour saturation during the diet manipulation differ among diet treatments. As post-hoc tests, we ran identical GLMM but with diet treatment as a 2-level factor (either control vs. lutein, or control vs. vitamin E).

A concern in this experiment is that, although there were 1-week intervals between the different diet treatments, there may be carry-over effects of one treatment on the bill colour saturation at the onset of the following treatment (i.e., effects of lutein supplementation extending to the beginning of the vitamin E treatment that follows it, or effects of vitamin E supplementation extending to the beginning of the control treatment that follows it). Therefore, to prevent possible carry-over effects from influencing analyses, we also run a GLMM using the measurements from the lutein or vitamin E diet treatments (average of weeks 2–4, as before) and, instead of the control treatment, the first bill colour measurement made at the beginning of the experiment, before any diet manipulation was made. In this GLMM, bill colour saturation is the dependent variable, individual identity is a random factor, and diet treatment is a 3-level factor (beginning, lutein, vitamin E).

For body weight, similarly to the main colour analysis, we performed a GLMM with body weight as dependent variable, individual identity as random factor, diet treatment (control, lutein, vitamin E) and time (as before, at the onset vs. during each diet manipulation) as factors, and the interaction between diet treatment and time. Again, the result of interest is the interaction effect, which tests if weight changed differently between treatments.

### Temperature experiment

#### Housing and temperature manipulation

This experiment took place in the same aviary as before, and lasted for 10 weeks, from the 10th December 2018 to the 20th February 2019, during the coldest months of the year. The waxbills used in this experiment were the same as in the previous experiment, except for one male that was added and one female that had died in the meantime, thus resulting in 12 males and 11 females. Birds were kept in mixed-sex groups throughout the experiment (two males and two females per cage, except one cage with two males and one female), and housing conditions were as described before.

Three of the bird cages received a cold–hot treatment, consisting of cold temperature (ambient temperature) for 4 weeks, then a 2-week interval, and hot temperature (heated cages) for other 4 weeks. The other three cages received the reverse treatment, hot–cold (hot temperature during the first 4 weeks, a 2-week interval without heaters, and then cold temperature during the final 4 weeks). This is a balanced design that cancels possible effects of confounding factors that may change through the year, such as seasonal variation in temperatures or in night duration. The mean ambient temperature during the first 4-week period was 11.2 °C (± 0.08 °C SD) and during the last 4-week period mean ambient temperature remained similar: 12.1 °C (± 0.04 °C SD; Figure S3). Four-week periods of temperature manipulation were previously shown sufficient to change bill colour in zebra finches (*Taeniopygia guttata*^16^). We heated cages by placing two warming devices (24 × 27.5 × 7 cm electric foot warmers, Junex model 5768, consisting of a 70 W warming plate protected by wooden frames) below each cage. In addition, the top 26 cm of the front grid of all cages was covered with transparent cling film, to retain warm air within the cage, and a 7 cm-high area in the bottom of the cage remained without cling film for aeration. The sides, back and top of each cage, made of metal, were covered on the outside by Styrofoam plates to further retain heat (Figure S2). For the onset of the hot treatment to be gradual, we turned on the warming devices on the first day of each 4-week period but did not immediately apply the cling film, which we then applied to all six cages (both heated or at ambient temperature) 2 days afterwards. In this manner, we could manipulate temperature differently across cages in the same shared environment, without having to isolate or move some of the birds to different rooms.

Each cage had a data logger (Elitech RC-5 USB Temperature Data Logger) hanging from the top of the cage, at the centre, recording temperatures every 30 min. Another temperature logger, located outside the cages in the aviary, also recorded temperature every 30 min. Throughout the experiment, the cold cages had the same temperature as outside in the aviary (ranging from 4.2 to 23.8 °C), and the temperature differences between these and the hot cages remained stable, averaging 5.7 °C (± 0.7 °C SD; Figure S3). Data loggers inside the cages failed to record temperatures during the second week of the experiments, and we replaced the missing data from that week by measurements from the external temperature logger plus the mean difference between temperatures inside and outside the cages, recorded during the remaining of the experiment (mean difference was 6.444 °C for the heated cages, and 0.679 °C for the ambient temperature cages). These reconstructed temperatures are represented by dashed lines in Figure S3.

#### Feeding, bill colour measurements and body weight

We set up a video system in the aviary room (two HP HD 2300 webcams connected to a computer) to automatically record the behaviour of the birds early in the morning, from half an hour before sunrise until 2 h later, on every Saturday, when there was no disturbance due to maintenance of the aviary. Videos were analysed by the same person (R.F.), using instantaneous observations at every 2 min along the 2 h of video, and noting how many birds were feeding (i.e., at a feeder or at less than one body of distance behind the feeders, ca. 10 cm). Video observations were made for the group in each cage, rather than for the individual birds, since the birds were not marked with colour rings, only numbered rings. We calculated an index of feeding in each cage, as the average number of birds feeding during the 2 h of observation.

We measured bill coloration in males and females throughout the experiment at eight time points: once at the end of each week of the two 4-week temperature manipulation periods (Figure S3). Measurements were taken always at ca. 2 p.m., first on the day when each 4-week temperature treatment began (before turning on the heating devices), and then once a week for the following 4 weeks of each period. Colour was measured using reflectance spectrophotometry, in the same way described above. We weighted birds with a spring balance to the nearest 0.05 g, by the end of each part of the experiment (9th January and 20th February) at the same time of the final colour measurements.

#### Statistical analyses

We computed changes in feeding, in body weight and in bill colour saturation as the difference between measurements in last and the first 4-week periods. We then tested how temperature manipulation affected changes in these traits by comparing birds in the cold–hot and the hot–cold treatments with independent samples *t* tests. In this way, and since the cold–hot and hot–cold treatments took place simultaneously, we test effects of temperature while controlling for longitudinal changes unrelated to the temperature manipulation. For comparing changes in feeding we used as statistical unit each of the cages (since observations of feeding were at the level of cages rather than individuals), and for comparing changes in body weight we used individuals as statistical units.

To test effects of the temperature manipulation on bill colour saturation we ran t-tests separately for males and females, and we computed changes in colour saturation in two different manners. First, we computed changes as the mean saturation across all measurements of the same individual in the last 4 weeks of the experiment minus the mean saturation across all its measurements in the first 4 weeks. Second, and in case bill colour takes a long time to change in response to temperature, we computed change as above but using only measurements from the last 2 weeks of each 4-week period. Additionally, we also tested if temperature manipulation affected the extent of fluctuation or stability in bill colour saturation. For this, we computed changes in bill colour fluctuation for each individual as the difference between the standard deviation of measurements in the last 4 weeks of the experiment minus the standard deviation of measurements in the first 4 weeks.

## Supplementary Information

Supplementary Information.
