# Supplementary material for: Ecological effects on female bill colour explain plastic sexual dichromatism in a mutually-ornamented bird
Source: Sci Rep. 2021 Jul 22;11:14970. doi: 10.1038/s41598-021-93897-z (PMC8298529; doi:10.1038/s41598-021-93897-z)
Supplement: Supplementary file 1 — Supplementary Information. [file 41598_2021_93897_MOESM1_ESM.pdf]

Ecological effects on female bill colour explain plastic sexual dichromatism in a mutually-ornamented bird

Rita Freitas, Cristiana Marques, Gonçalo C. Cardoso, Sandra Trigo

Supplementary Figures and Supplementary Tables

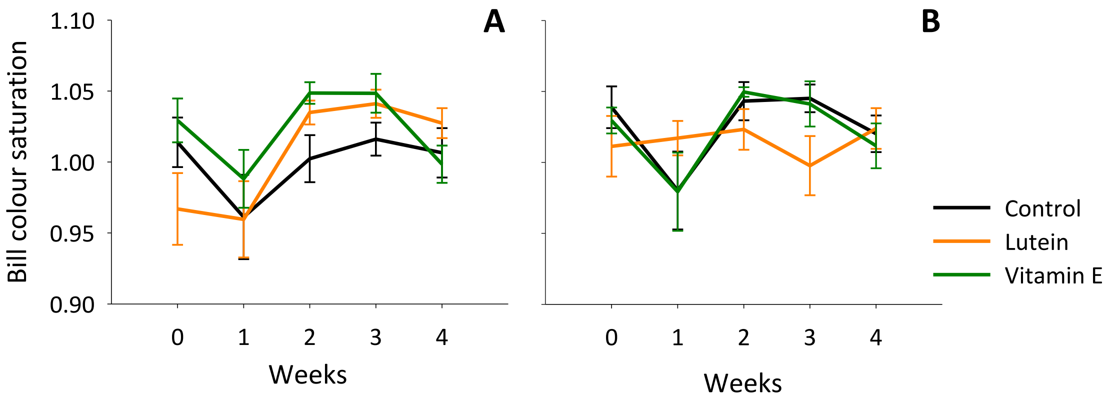

Figure S1. Bill colour saturation of (A) females and (B) males (mean ± SE) during each 4-week diet manipulation period.

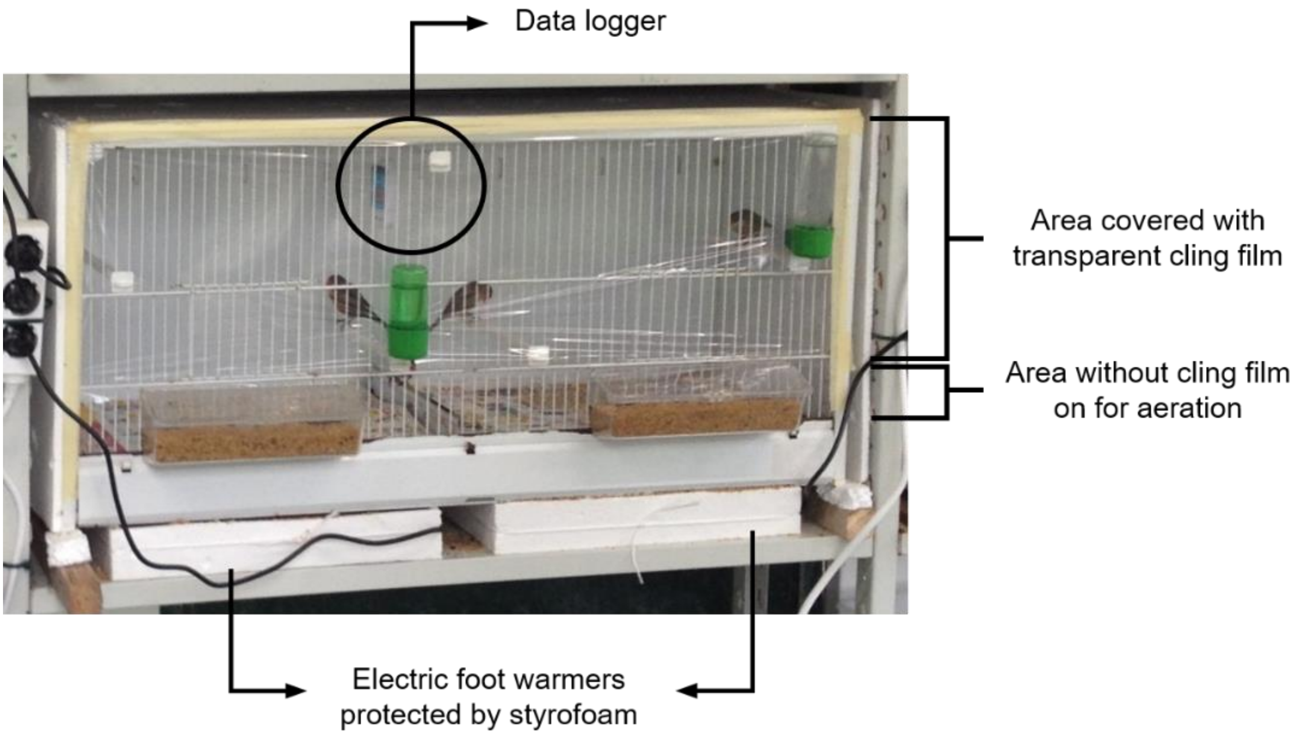

Figure S2. Setup of the bird cages in the temperature manipulation experiment.

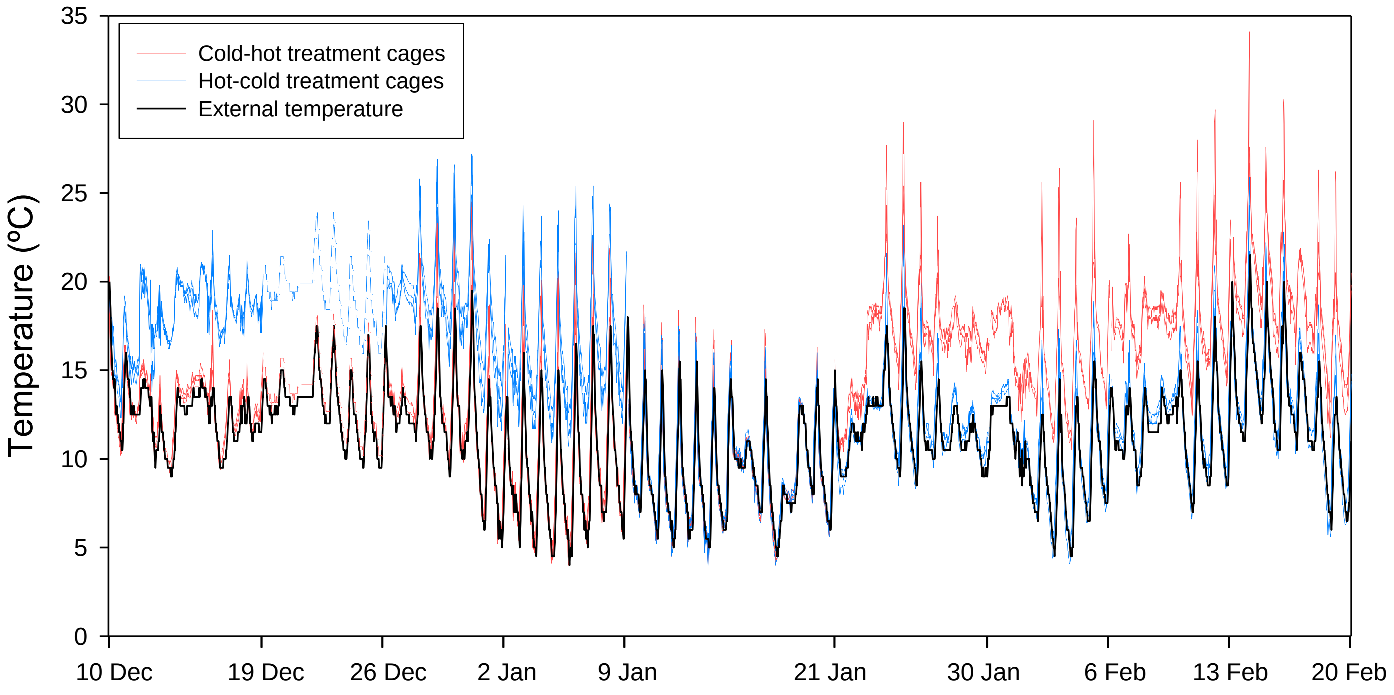

Figure S3. Fluctuations in measured ambient temperatures along the experiment outside (black line) and inside each bird cage (3 nearly identical red lines, and 3 nearly identical blue lines). Dashed portions of the lines, during one week in December, correspond to the reconstructed temperatures inside the cages (see 'Materials and methods'). Dates written along the time axis mark the onset of temperature manipulations and each of the bill colour measurements.

Table S1. Post-hoc tests for the effects of diet manipulation on bill colour saturation for females. Results of a GLMM with bill colour saturation as dependent variable, individuals as subjects, treatment and date as factors, and their interaction. N = 12

	**Control vs. Lutein**		**Control vs. Vitamin E**	
Before vs After	*F*	*P*	*F*	*P*
Intercept	14273.30	0.000	22338.17	0.000
Treatment	0.38	0.540	0.161	0.161
Date	3.38	0.073	0.013	0.911
Treatment * Date	4.71	**0.035**	0.768	0.768

Table S2. Effects of diet manipulation on bill colour saturation for females (N = 12) and males (N = 11). Results of a GLMM with bill colour saturation as dependent variable, and different treatments as factor (initial values and after values for control, lutein and vitamin E treatments).

	**Females**		**Males**	
	F	*P*	*F*	*P*
Intercept	22234.68	0.000	38400.24	0.000
Treatment	6.55	**0.001**	1.01	0.398
